# Exploration of biomarkers for the diagnosis, treatment and prognosis of cervical cancer: a review

**DOI:** 10.1007/s12672-022-00551-9

**Published:** 2022-09-24

**Authors:** Masita Arip, Lee Fang Tan, Rama Jayaraj, Maha Abdullah, Mogana Rajagopal, Malarvili Selvaraja

**Affiliations:** 1 Allergy & Immunology Research Centre, Institute for Medical Research, National Institute of Health, Setia Alam, 40170 Shah Alam, Selangor Malaysia; 2 Department of Pharmaceutical Biology, Faculty of Pharmaceutical Sciences, UCSI University, 56000 Cheras, Kuala Lumpur, Malaysia; 3 Immunology Unit, Department of Pathology, Faculty of Medicine and Health Sciences, Universiti Putra Malaysia, Jalan Serdang, 43400 Serdang, Selangor Malaysia; 4 Charles Darwin University, Darwin, NT 0909 Australia

**Keywords:** Biomarker, Cervical cancer, Omics, Genomics, Transcriptomics, Proteomics, Metabolomics, High throughput

## Abstract

As the fourth most diagnosed cancer, cervical cancer (CC) is one of the major causes of cancer-related mortality affecting females globally, particularly when diagnosed at advanced stage. Discoveries of CC biomarkers pave the road to precision medicine for better patient outcomes. High throughput omics technologies, characterized by big data production further accelerate the process. To date, various CC biomarkers have been discovered through the advancement in technologies. Despite, very few have successfully translated into clinical practice due to the paucity of validation through large scale clinical studies. While vast amounts of data are generated by the omics technologies, challenges arise in identifying the clinically relevant data for translational research as analyses of single-level omics approaches rarely provide causal relations. Integrative multi-omics approaches across different levels of cellular function enable better comprehension of the fundamental biology of CC by highlighting the interrelationships of the involved biomolecules and their function, aiding in identification of novel integrated biomarker profile for precision medicine. Establishment of a worldwide Early Detection Research Network (EDRN) system helps accelerating the pace of biomarker translation. To fill the research gap, we review the recent research progress on CC biomarker development from the application of high throughput omics technologies with sections covering genomics, transcriptomics, proteomics, and metabolomics.

## Introduction

Despite being highly preventable, cervical cancer (CC) is the fourth most common gynecological malignancy threatening women health and lives due to the insufficient screening protocols, particularly in low‑ and middle‑income countries [1–3]. According to the World Health Organization (WHO), it is estimated that in 2018, approximately 570,000 cases of CC were diagnosed and about 311, 000 females died from the disease [4]. Persisting infection with high-risk subtypes of the human papilloma virus (hrHPV) is the main cause of cervical carcinogenesis [5].

Asymptomatic and non-specific nature in the early stages of CC often lead to late-stage diagnosis [6]. Cytology-based screening, known as Papanicolaou test (Pap smear) and HPV testing are the most frequently used methods for CC screening in the clinical practice [7]. However, the current screening programs have some limitations such as causing patient discomfort, the invasive and sensitive nature of the tests, as well as low levels of sensitivity and specificity. Early detection of disease is extremely important due to the availability of various treatment options which make CC curable [8]. The treatment options available for CC are surgery, radiation, chemotherapy, or in a combination, which may cause various side effects and no cure[9, 10]. The poorer prognosis and ineffective treatment in the advance stage of CC necessitate the development of new prognostic, diagnostic, and therapeutic strategies [11, 12].

A cancer biomarker is a substance or process indicative of the presence of cancer, which can be secreted by a malignancy itself, or as a specific body response to the presence of cancer [13]. The discovery of biomarkers including genes, DNA, RNA, proteins, enzymes, antigens, and other cellular and biological products paves the road to precision medicine for better patient outcomes through the classification of patients by probable disease risk, treatment and prognosis [14]. Thus, identification of CC biomarkers is expected to provide greater direction in strategizing the prevention and treatment of CC [15]. Various biomarkers concerning carcinogenesis, precancerous lesions, and CC have been described in many articles and reviews [15]. For instance, the well-known markers P16 and Ki-67 have demonstrated promising results as surrogate biomarkers of cervical neoplasia [16–18]. A recent meta-analysis confirmed that p16 and p16/Ki-67 immunocytochemistry has higher specificity for cervical intraepithelial neoplasia of grade 2 or worse (CIN2+) or cervical intraepithelial neoplasia of grade 3 or worse (CIN3+) than the hrHPV DNA testing [16]. Similar sensitivity was reported for dual staining and the hrHPV DNA testing. The application of p16/Ki-67 dual-stained cytology for detection of cervical precancer and cancers in various settings may limit the burden of over-detection such as unnecessary health care costs and potential adverse events due to overtreatment [16].

Omics technologies focused at the universal detection of genes (genomics), mRNA (transcriptomics), proteins (proteomics) and metabolites (metabolomics) in a biosample have revolutionized medical research [19]. It is possible to gather vast amounts of data of a particular type of molecules in a single experiment through these high throughput technologies [20]. A remarkable growth in the assay technologies which includes single nucleotide polymorphisms (SNP) arrays, gene expression microarrays and protein arrays continue to identify various novel biomarkers aimed for precision medicine [21]. Multi-omics approaches integrating omics data across different levels of cellular function enables better understanding of the molecular and clinical features of the disease, contributing to enhanced ability to address applications including disease subtyping and biomarker prediction [22].

To date, researchers have highlighted numerous biomarkers offering new prospects for translational CC research, however the focus on the contributions of high throughput omics technologies towards the process of CC biomarker development has not been extensively discussed [23–26]. Hence, the present review aims to summarize the various biomarkers associated with diagnosis, treatment and prognosis of CC discovered in the past five years through omics technologies at the aspects of genomics, transcriptomics, proteomics and metabolomics for precision medicine.

## Methodology

**Fig. 1:**
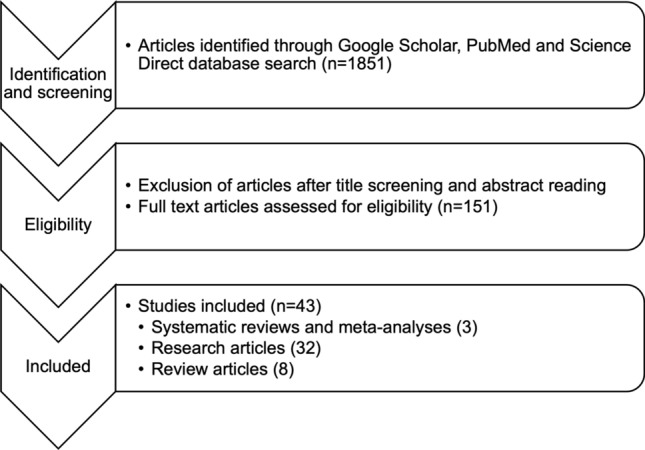
Schematic representation of selection of articles for the review

This article is a general descriptive review summarizing various CC biomarkers discovered through high throughput omics technologies with most data cited ranging January 2016 to August 2021 for the most recent published study. A search was performed using online databases including Google Scholar, PubMed and Science Direct using search words and strings, mainly “cervical cancer”, “biomarker”, “omics”, “genomics”, “transcriptomics”, “proteomics” and “metabolomics”. Selection of articles was summarized in Fig. 1. Studies (original, review, systematic, meta-analysis) covering the following types of data were included and extracted: application of high throughput omics technologies with sections covering genomics, transcriptomics, proteomics and metabolomics as well as biomarkers associated with diagnosis, treatment and prognosis of CC. Studies were excluded if written in other languages than English due to language barrier.

## Significance of biomarkers for CC

Accurate and predictable early screening of CC is crucial [27]. Although Pap smear can easily detect squamous lesions, it cannot detect glandular lesions as such lesions are only visible in histological examination via biopsy. On the other hand, although hrHPV DNA testing has become an important tool, the tests are limited by low specificity and inability to predict the infection outcome. Biomarkers may be implemented in various steps within the disease flowchart. The identification of biomarkers for CC will help to diagnose the conditions at early stage of disease development and help to control the condition from progressing to severe stage [8]. Utilization of biomarkers may help in making timely clinical management decisions such as further testing, treatment, colposcopy referral, increased surveillance or release to routine screening [27]. Biomarkers can also be applied to estimate the prognosis of patients, to determine the treatment impact, and to monitor the treatment progression. Biomarkers play a role in the development of precision medicine as the treatments to individual or subgroups of patients can be adjusted based on specific biomarkers for optimal patient outcomes [28].

## Results and discussion

### CC biomarkers discovered through genomics

Genomic markers causing genetic alterations have roles in the carcinogenesis and progression of CC. Genome-wide association studies (GWAS) and next generation sequencing (NGS) are the omics technologies widely used to investigate the genetic risk factors and mutation profiles in tumors, including CC [20, 29].

Numerous studies reported on the effect of SNPs on CC susceptibility [30, 31]. Heritability may be used to quantify the proportion of CC predisposition attributable to host genetic factors and it was estimated that shared genes account for 27% of CC heritability [32]. Human leukocyte antigen (HLA) genes exhibit statistically significant associations at the locus 6p21.3 (HLA class I and II genes) and two loci outside HLA at 4q12 (EXOC1), and 17q12 (GSDMB) [32–34]. With the lead SNP rs59661306 and rs7457728, novel-significant associations were identified at 5q14 and 7p11 respectively. Functional studies using cervical HeLa cell lines suggested the role of ARRDC3 gene in cell growth and susceptibility to HPV infection [35]. Disruption in apoptotic and immune function pathways at PAX8 and CLPTM1L and interaction between TP53 and XRCC1 increases the genetic susceptibility to CC [36]. The difficulty in interpreting GWAS associations limits the translation of the findings into clinical care [37, 38]. There have been concerns that the whole genome will be implicated in the disease predisposition and that the variants and genes reflected in association signals show no direct biological linkage to the disease [38]. Most of the disease-associated loci lie in the non-coding regions of the genome with regulatory role, questions regarding the genes regulated and cell types or physiological contexts the regulation occurs arise [37].

The persistent infection with hrHPV causes viral integration into the host genome up to 76.3% of CC cases with positive correlation to CIN grades [39], which can be detected with NGS. HPV integration, the key genetic mechanism reported at least 83% of HPV-associated CC commonly occur at particular fragile sites [40], significantly upregulate the gene expression and it has been associated with poorer rate of survival compared to those with episomal form of HPV. Therefore, the HPV integration status may consider as a promising biomarker for diagnosis, risk stratification, therapy, prediction of treatment responses and treatment monitoring [39, 40]. Analysis of blood samples with NGS technologies demonstrated the potential use of RNF213 mutation as a biomarker to monitor the treatment response to chemotherapy and radiotherapy [41]. Although NGS allows the whole sequence of cancer’s exome or genome to be obtained, not all information provided contribute substantially to the determination of the clinical decisions for cancer patients, for which smaller targeted sequencing panels are often more clinically practical [42]. NGS is also limited by the need for extensive analytic capabilities which may be costly. Other limitations include difficulties in identifying the driver mutations and confounding factor of tumor heterogeneity [43]. Table 1 shows the summary of studies on CC biomarkers discovered through genomics.

**Table 1:** Summary of studies on CC biomarkers discovered through genomics

Article type	Population	Study period	Sample size	Source of sample	Platform/assay technique	Stage of research	Association to CC	Significance	References
Research article	Taiwan	2017	507 CSCC cases 432 age/sex matched healthy controls	Cervical tissue	PCR	Case control study	Protective marker/ decreased risk	Genotype G/T and allele G of SNP rs4282438 rs4282438 SNP (OR = 0.67, 95% CI 0.55–0.80)	[29]
Research article	China	2016	121 CC cases 118 healthy controls 101 elderly patients aged > 80 (no CC history)	Peripheral blood	MAMA-PCR	Case control study	Risk/susceptible marker	Mutation of XRCC1 rs25487 2-locus SNP-SNP interaction pattern (XRCC1 rs25487 and TP53 rs1042522) with CC risk (cases vs negative controls: OR = 4.63, 95% CI = 1.83–11.75; cases vs elderly group: OR = 17.61, 95% CI = 4.34–71.50)	[44]
Research article	India	NA	63 HPV16 + cases 61 HPV16 + non-tumors 41 HPV- controls	Tissue	Real-time PCR	Case control study	Risk/ susceptible marker Protective marker	HLA-B*40:06 in CC cases (OR = 5.178, 95% CI = 1.856–14.451) and asymptomatic infection (OR = 3.954, 95% CI = 1.610–9.706) HLA-B*15:02 (protective SNP-based signature, GAATTTA) in CC (OR = 0.117, 95% CI = 0.029–0.470) and asymptomatic infection (OR = 0.163, 95% CI = 0.043–0.623)	[30]
Research article	Saudi Arabia	1990–2012	232 ICC cases 313 healthy controls	Blood	Direct sequencing HPV linear array analysis	Case control study	Protective marker	TP53 G72C genotype with HPV positivity (OR = 0.57, 95% CI = 0.36‐0.90) Variant C allele in low CC incidence population	[31]
Research article	European	1999–2010	2866 cases 6481 controls	NA	BeadArray technology	Case control study	Risk/ susceptible marker	(HLA-DRB1*1501/HLA-DQB1*0602/HLA-DQA1*0102, HLA-DRB1*0401/HLA-DQA1*0301) and protective (HLA-B*15, HLA-DRB1*1301/HLA-DQB1*0603/HLA-DQA1*0103) HLA haplotypes, depending on the risk or protective amino acids at positions 13 and 71 in HLA-DRB1, and position 156 in HLA-B	[32]
Research article	East Asian	1996–2005	2609 cases 4712 controls	Tissue, serum	BeadArray technology MassARRAY	Case control study	Risk/ susceptible marker	Associations at 5q14 using lead SNP rs59661306 (p = 2.4 × 10^–11^) and at 7p11 with rs7457728 (p = 1.2 × 10^–8^) In 5q14, the chromatin region of GWAS-significant SNPs was in contact with the ARRDC3 promoter ARRDC3 in HPV entry demonstrated by markedly decreased cell growth and susceptibility to HPV16 pseudovirion infection resulted from ARRDC3 knockdown in HeLa cells	[35]
Research article	European	2006–2010	4769 CIN3 and ICC cases 145 545 controls	Tissue	Microarray	Case control study	Risk/susceptible marker	rs10175462 (PAX8; OR = 0.87, 95% CI = 0.84–0.91), rs27069 (CLPTM1L; OR = 0.88, 95% CI = 0.84–0.92), rs9272050 (HLA-DQA1; OR = 1.27, 95% CI = 1.21–1.32), rs6938453 (MICA; OR = 0.79, 95% CI = 0.75–0.83), rs55986091 (HLA-DQB1; OR = 0.66, 95% CI = 0.60–0.72), and rs9266183 (HLA-B; OR = 0.73, 95% CI = 0.64–0.83) with CIN3 and ICC	[36]
Research article	Korea	2017	24 CC cases	Blood	NGS	Prospective cohort study	Monitoring marker to response to chemo- and radiotherapy	75% of the samples showed mutations including ZFHX3, KMT2C, KMT2D, NSD1, ATM and RNF213, with RNF213 mutation	[41]

### CC biomarkers discovered through transcriptomics

Microarrays and RNA sequencing (RNA-Seq) employs high throughput sequencing to capture the sequences of the whole transcriptome are the two key techniques used for transcriptome study [45]. Compared with microarrays, the identification of more differentially modulated transcripts, splice variants, and non-coding transcripts with higher fold-change by RNA-Seq technology provides additional data that may be informative for clinical prediction, mechanistic investigations or biomarker discovery [46, 47].

The noncoding RNAs (ncRNAs) are known as oncogenic drivers and tumor suppressors in CC [48, 49]. Epigenetic modifications including deregulated expression of ncRNAs and circular RNAs (circRNAs) involve in the initiation and promotion stages of CIN and cervical carcinoma [49]. MicroRNAs (miRNAs), long noncoding RNAs (lncRNAs) and circRNAs have also been associated with CC metastasis through the regulation of related genes, epithelial-mesenchymal transition, signaling pathways and interactions with microenvironment of tumors [50].

Small, single stranded miRNAs are the master modulators of genome which regulate up to 60% of protein-coding genes and they are involved in processes such as cell cycle regulation, differentiation, programmed cell death, angiogenesis, DNA repair or stress response [51]. Altered miRNAs can roughly be classified as oncogenic and oncosuppressor miRNAs, and both have been correlated with biological processes in CC progression [52]. Expression miR-29a and miR-21 are reported as the most frequently down- and up-regulated miRNAs respectively in the progression of invasive CC [53]. However, there was a small overlap between the results of microarray-based studies, with miR-10a, miR-20b, miR-9, miR-16 and miR-106a was found to be upregulated, whereas miR-99a, miR-203, and miR-195 were reported to be down-regulated [53]. Differences in study designs, populations, arrays used, convenience material-based studies and small sample size may be the plausible explanations for the variations. Improved performance has been reported with the combined use of miRNA markers [54]. A combination of six upregulated oncogenic miRNAs (miR-20a, miR-92a, miR-141, miR-183*, miR-210 and miR-944) showed enhanced accuracy for diagnosis of CC compared with individual use of any marker with an excellent AUC of 0.959, sensitivity of 91.4%, and specificity of 87.6% [55]. Cervical adenocarcinoma has been reported to be associated with higher rate of metastasis and treatment resistance than squamous cell carcinoma. Through transcriptome analysis, study reported the improved diagnostic performance for cervical adenocarcinoma from the combination of miR-192-5p, HNF1A-AS1, and VIL1 with an AUC of 0.911, which could be promising diagnostic biomarkers for cervical adenocarcinoma [56].

As miRNAs, the crucial roles of lncRNAs in cell growth, survival, cell cycle, differentiation and apoptosis have been demonstrated and their roles as molecular regulatory factors in CC may provide opportunities for early diagnosis and therapeutic targets to improve clinical outcomes [57, 58]. lncRNA microarray analysis revealed the oncogenic lncRNA‑AK001903 which promotes tumor progression in CC [59]. Transcriptomic and lncRNA-mRNA correlation analysis showed PCBP1-AS1 as a novel prognostic biomarker for CC. The elevated expression of PCBP1-AS1 is associated with tumor stage, TNM and invasion [60]. A recent study integrating the data of DNA methylation, copy number variation (CNV) and transcriptome to identify CNV-related lncRNAs for CC prognosis prediction have developed a 8-lncRNA (RUSC1-AS1, LINC01990, LINC01411, LINC02099, H19, LINC00452, ADPGK-AS1, C1QTNF1-AS1) signature with high AUC independent of clinical features, providing novel prognostic biomarkers for CC [61].

The differential expression of circRNAs in CC cells compared with normal cells suggests their potential roles and biological relevance in CC. CDR1as is one of the most well-identified circRNAs which sponges miRNA‐7, a tumor suppressor that has been associated with CC [62]. In vitro studies to investigate the roles of circRNAs in cervical carcinogenesis and progression reported upregulated circRNAs such as has_circ_0018289 (miR-497 sponge), has_circ_0018289 (miRNA-497 sponge), has_circ_0023404 (miRNA-136 sponge), has_circ_0000263 (miRNA-150-5p sponge), circRNA‐000284 (miRNA-506 sponge), has_circRNA_101996 (miRNA‐8075 sponge), circ‐ATP8A2 (miRNA‐433 sponge), circ_0067934 (miRNA‐545 sponge), circEIF4G2 (miRNA‐218 sponge) and circRNA8924, while the has_circ_0001445 (miRNA‐620 sponge) has been found to be downregulated [63]. The expression abundance, stability and specificity conferred by circRNAs make them as potential biomarker for cancers but further studies required as studies of circRNAs in CC, particularly their mechanisms of action are still at the nascent stage [63, 64].

Combined differential expression and differential co-expression analysis revealed, epidermis development-associated gene set around ZNF135 act as putative biomarker for the prevention and treatment of CC [65]. More recently, five out of the seven co-expressed gene modules identified by differential co-expression network analysis were reported to exhibit high capabilities for diagnosis and prognosis [11]. These gene modules were associated with biological processes including regulation of cell cycle, keratinization, degranulation of neutrophils as well as phospholipase D signaling pathway. AR, E2F4, ESR1, ETS1, FOXP3, GATA1, GATA2, GATA3, PRDM14, and YBX1 were the transcription factors regulating the module genes and ETS1 and GATA2 were found as the common regulatory elements in most modules. The incorporation of differential co-expression analyses in the search of molecular basis of complex diseases recommended to achieve systems-level understanding of the variation in disease phenotype in CC [11].

All low [e.g., in situ hybridization (ISH), subtractive hybridization (SH), Northern Blot (NB), ribonuclease protection assay (RPA), reverse transcription-polymerase chain reaction (RT-PCR)], medium- [e.g., expressed sequence tags (EST), Open Reading frame ESTs (ORESTES)] and high throughput [e.g. microarrays, serial analysis of gene expression (SAGE) and massively parallel signature sequencing (MPSS)] techniques have their pros and cons, with high throughput methods are characterized by big data production whereas low throughput methods offer higher specificity, sensitivity, and reproducibility [66, 67]. With that, there is a need for the high- and medium-performance techniques to be validated by low-performance techniques [66]. Combination of miRNA signatures with other different markers may help to improve risk stratification. Table 2 shows the summary of studies on CC biomarkers discovered through transcriptomics.

**Table 2:** Summary of studies on CC biomarkers discovered through transcriptomics

Article type	Population	Study period	Sample size	Source of sample	Platform/ assay technique	Stage of research	Association to CC	Significance	References
Review article	NA	2011–2020	NA	NA	High throughput sequencing technology	NA	Prognostic marker, CC metastasis	Dysregulation of ncRNAs miRNAs, miR-21, miR-221-3p, miR-199b-5p, miR-29a, miR-543, miR-106b, miR-519d, miR-218-5p, miR-200b, miR-484, miR-145, miR-211, miR-183, miR-124 and miR-221-3p lncRNAs, MALAT1, EBIC, TUG1, CTS, HOTAIR, Xist, 799, XLOC_006390, TTN-AS1, ZNF667-AS1, DANCR, PVT1, GA5-AS1, DGCR5 and ANRIL circRNAs, circ-0000745, circ-000284, circ-NRIP1, circ-0003204 and circUBAP2	[50]
Systematic review	NA	2010–2017	24 studies	Tissue	RT-PCR qPCR Microarray	NA	Risk/ susceptible marker Prognostic biomarker	Downregulation of miR-29a and upregulation of miR-21	[53]
Research article	Hong Kong	2006–2013	582 cases 145 controls	Tissue	qPCR	Multiphase case–control study	Diagnostic marker	Upregulation of miR‐20a, miR‐92a, miR‐141, miR‐183*, miR‐210 and miR‐944	[55]
Research article	China	2009–2018	165 cervical adenocarcinoma cases 81 normal controls	Tissue	RT-qPCR	Case control study	Diagnostic marker	Upregulation of VIL1, HNF1A-AS1, MIR194-2HG, SSTR5-AS1, miR-192-5p, and miR-194-5p in adenocarcinoma Combined miR-192-5p, HNF1A-AS1, and VIL1	[56]
Review article	NA	2007–2016	NA	NA	NA	NA	Diagnostic marker Prognostic marker Therapeutic marker	HOTAIR, MALAT1, CCAT2, SPRY4-IT1, RSU1P2, CCHE1, lncRNA-EBIC and PVT1	[57]
Research article	China	2016–2017	29 CC tissues and peritumoral tissues	Tissue	Microarray RT-qPCR	Case control study In vitro (cell lines)	Prognostic marker	Upregulation of lncRNA-AK001903	[59]
Research article	China	2012–2021	23 pairs of CC and adjacent tissues	Tissue	Microarray qPCR Western blot	Case control study	Prognostic marker	Elevated PCBP1-AS1	[60]
Research article	NA	NA	292 CC specimens	NA	iClusterPlus DESeq2 GSEA WGCNA GSVA	Case control study	Prognostic marker	lncRNAs-based signature consisted of 8 lncRNAs, namely RUSC1-AS1, LINC01990, LINC01411, LINC02099, H19, LINC00452, ADPGK-AS1, C1QTNF1-AS1	[61]
Review article	NA	2003–2019	NA	NA	NA	NA	Diagnostic marker Therapeutic marker	circRNAs in CC carcinogenesis and progression	[63]
Research article	China	2015–2017	352 CC cases 204 CIN cases 227 healthy controls	Tissue	RT-PCR Western blot	Case control study In vitro (cell lines)	Diagnostic marker Prognostic marker	Elevated CDR1	[68]
Research article	NA	NA	87 CC samples 44 normal controls	NA	Differential expression analysis using t-test Differential co-expression analysis using Fisher Z- test	Case control study	Risk/susceptible marker Therapeutic marker	Epidermis development-associated gene set around ZNF135	[65]

### CC biomarkers discovered through proteomics

Protein microarrays, mass spectrometry (MS) and nuclear magnetic resonance (NMR) spectroscopy are some of high throughput techniques used in proteomics to determine protein expression levels which could not be achieved by conventional techniques such as one-dimensional SDS–polyacrylamide gel electrophoresis (1D SDS-PAGE) gels, Western Blot or enzyme-linked immunosorbent assay (ELISA) [69–71].

Membrane proteomics of one normal cervical (HCK1T) and there cervical cell lines, C33A (HPV-negative), SiHa (HPV16+), HeLa (HPV18+) have revealed the differentially expressed membrane proteins which are involved in cancer-associated biological pathways such as HIPPO, PI3K/Akt s and EIF2 signaling as well as cell cycle G2/M DNA damage checkpoint regulation which may be putative markers for diagnosis, prognosis and treatment [72]. Intracellular proteomics of the four cell revealed the upregulation of cofilin-1 [73]. Inhibition of matrix metalloproteases in cancer cell lines was found via secretome analysis of the cell lines, and this was further validated by zymography for MMP-2 and MMP-9, western blot analysis for ADAM10, CATD, FUCA1 and SΟD2, and multiple reaction monitoring (MRM) for CATD, CATB, SOD2, QPCT and NEU1 [74]. The biochemical similarities and differences among the four representative and informative cell lines reflect the aberrant pathways involved in cervical carcinogenesis, providing valuable information for the identification of biomarkers of cervical pathology [73].

Various protein markers have been identified through the proteomic analysis using biological samples including serum, cervical mucus, cervicovaginal fluid (CVF) and urine [8, 75–81]. Non-invasive measurement of tumor biomarkers in serum such as carcinoembryonic antigen (CEA), squamous cell carcinoma antigen (SCC-Ag) and carbohydrate antigen 19-9 (CA19-9) have been frequently employed in CC detection and monitoring but their specificity for CC detection and sensitivity for early stage detection are of unsatisfactory levels [8]. The significantly elevated levels of serum SCC-Ag, highly sensitive C-reactive protein (hs-CRP), and CA-125 in recurrence cervical patients indicates that these proteins could be potential biomarkers for the prediction of recurrence risk [75]. Vascular endothelial growth factor (VEGF) is the main mediator of angiogenesis which stimulates the formation of new blood vessels, contributing to tumorigenesis and cancer progression. It has been reported to be overexpressed in 63.07% of patients with cervical carcinoma compared to controls and it is associated with poor prognosis [76]. A recent meta-analysis concluded elevated expressions of VEGF and VEGF-C were significantly associated with poor survival outcome in patients with CC [77]. Angiopoietins also play important roles in angiogenesis. Serum angiopoietin 2 (sAng-2) and the ratio of sAng-1/sAng-2 reported as potential diagnostic and prognostic biomarkers in CC [78]. A non-targeted proteomic analysis of cervical mucus profiled the differently expressed proteins in cervical adenocarcinoma, including heme protein myeloperoxidase and apolipoprotein A–I (APOA1), which play roles in immune response and lipid metabolism respectively [79]. Self-sample collection of cervical tissue using brushes, tampons, swabs or lavages for subsequent DNA genotyping, cytology or immunohistochemistry is a good method to be considered for screening purpose. CVF which can simply be collected in a non-invasive manner offers new opportunities for the development of self-tests. Functional classification of CVF proteome using proteomics technologies shows various biological roles, particularly protein metabolism and modification as well as immunity and defense [80]. Alpha-actinin-4 (ACTN4) is one of the proteins in CVF found to be a promising biomarker for the development of a simple assay for self-screening of cervical (pre)cancer [80]. Urinary samples is another source of biomarkers that can be easily and non-invasively obtained. Study with urines reported a significant upregulation of leucine rich α 2 glycoprotein (LRG1) and isoform 1 of multimerin 1 (MMRN1), and downregulation of S100 calcium-binding protein A8 (S100A8), SERPINB3 and cluster of differentiation-44 antigen (CD44) in CC. Through the receiver operator characteristic curve (ROC) analysis, the combination of these proteins or individual use of LRG1 and SERPINB3 may be detection biomarkers for CC [81].

The high throughput technologies used in proteomics studies are still relatively old and the limitations in protein quantification, data collection, sensitivity and reproducibility restrict the discovery of clinically significant novel biomarkers [82]. Significant differences in type of biomarkers identified and concentration reported exist across the results reported, even with the use of same biological samples. Integration of information generated from proteomics and validation of proteins that have been identified as potential biomarkers may accelerate the development of individualized patient care through clinical proteomics [83]. Table 3 shows the summary of studies on CC biomarkers discovered through proteomics.

**Table 3:** Summary of studies on CC biomarkers discovered through proteomics

Article type	Population	Study period	Sample size	Source of sample	Platform/assay technique	Stage of research	Association to CC	Significance	References
Research article	NA	2011–2014	86 cases	Serum	ELISA	Case control study	Risk/susceptible marker Prognostic marker	Elevated serum SCC-Ag, hs-CRP, and CA-125	[75]
Research article	China	2012–2014	77 CC patients 44 CIN patients 43 controls	Serum	ELISA	Non-matched case control study	Diagnostic marker Prognostic marker	Gradual increase of sAng-2 concentration from normal control Decreased sAng-1/sAng-2 Potential roles of sAng-2 and sAng-1/sAng-2 ratio	[78]
Meta-analysis	NA	2000–2011	1306 patients	Serum, tissue	IHC ELISA RT-PCR	In vivo (clinical trial) Case control study	Prognostic marker	Over-expressed VEGF and VEGF-C	[77]
Research article	Sudan	NA	65 cervical carcinoma cases 10 inflammatory lesions samples (controls)	Tissue	IHC	Case control study	Prognostic marker	VEGF and Her-2	[76]
Research article	Thailand	2014–2015	24 urine samples from CC patient 13 urine samples from HPV-negative females	Cells, urine	LC–MS/MS Western blot	Case control study	Diagnostic marker	Upregulated urinary proteins of LRG1 and MMRN1 and downregulated S100A8, SERPINB3 and CD44	[81]
Research article	China	2015–2019	200 cases 200 healthy controls	Peripheral blood	Immunoassay	Case control study	Diagnostic marker	miRNA-29a, miRNA-25, miRNA-486-5p with SCC Ag	[8]
Research article	China	NA	3 normal controls (Ctrl) 3 EA 3 cervical AIS	Cervical mucus	LC–MS IHC	Case control study	Diagnostic marker Therapeutic marker	237, 256 and 242 differently expressed proteins in EA/Ctrl, AIS/Ctrl and AIS/EA comparison	[79]

### CC biomarkers discovered through metabolomics

Various studies have been conducted comparing the metabolomics profiles of blood, urine, cervicovaginal lavage and tissue samples in identifying diagnostic, predictive or prognostic biomarkers [84–89]. From the plasma metabolomics conducted using ultra-performance liquid chromatography-quadrupole-time-of-flight mass spectrometry (UPLC-QTOF-MS) combined with multivariate statistical analysis, five differential metabolites including bilirubin, LysoPC(17:0), n-oleoyl threonine, 12-hydroxydodecanoic acid and tetracosahexaenoic acid were identified as the candidate biomarkers for CC with the area under curve (AUC) of 0.99 [84]. Phosphatidyl choline (15:0/16:0), phosphatidyl glycerol (12:0/13:0), actosylceramide (d18:1/16:0), D-Maltose, and phthalic acid with an AUC greater than 0.75, were pinpointed as potential prognostic biomarkers for cervical squamous cell carcinoma (SCC) by Zhou et al. through plasma metabolomics [85]. Another plasma metabolomics for diagnostic algorithm by Khan et al. [86] reported seven metabolites (adenosine monophosphate, aspartate, glutamate, hypoxanthine, lactate, proline, and pyroglutamate) which distinguished patients with CINs and CC from the healthy controls (AUC = 0.82 and 0.83 respectively). Metabolomics analysis of the urine samples using GC–MS to discriminate the HPV categories between patients revealed the closer metabolome of HPV + B (HPV positive with concomitant low and high-risk infections) with HPV − (HPV negative) than to HPV + H (HPV positive exclusively high-risk), suggesting the antagonism of HPV co-infections resulting from viral interference. Three urinary metabolites 5-oxoprolinate, erythronic acid (AUC = 0.92) and N-acetylaspartic acid (AUC = 0.91) identified differentiate those with HPV + H from the negative controls [87]. Metabolic analysis of cervicovaginal lavage revealed membrane lipids (3-hydroxybutyrate, eicosenoate, and oleate/vaccenate with excellent discrimination capacity AUC > 0.9) discriminated the invasive cervical carcinoma patients with the healthy controls and membrane lipids including sphingolipids, plasmalogens, and linoleate were positively correlated with genital inflammation. Non-*Lactobacillus* dominant communities resulted in perturbed metabolisms of amino acid and nucleotide, especially in high-grade dysplasia, connecting vaginal dysbiosis to cervical dysplasia, hence cervicovaginal metabolome may be a potential target for clinical interventions [88]. Tissue-based metabolomics to identify diagnostic biomarkers for HPV-associated cervical carcinoma showed decreased levels of α- and β-glucose, elevated levels of lactate and low-density lipoproteins as well as altered amino acid expression in HPV16-positive SCC or its precursor lesions compared with HPV-negative negative controls. The significantly upregulated expression of glycogen synthase kinase 3 beta (GSK3β) and glutamate decarboxylase 1 (GAD1) and decreased for pyruvate kinase muscle isozyme 2 (PKM2) and carnitine palmitoyltransferase 1A (CPT1A) in cervical lesions imply that increased aerobic glycolysis and disrupted lipid metabolism may confer advantages for tumor growth [89].

Although metabolomics has shown high potential in hypothesis generation and biomarker discovery, numerous challenges have to be addressed for the advancement of this relatively new omics field [90]. Difficulty in replicating the metabolomic biomarkers across various studies may be attributed to sample sources, population heterogeneity, experimental protocols, data parameter setting biological variations in metabolite turnover rates, thus limiting the application of novel cancer biomarkers in clinical settings [84]. Integrating metabolomics with other omics data may help to achieve improved translational outcomes [91]. Table 4 shows the summary of studies on CC biomarkers discovered through metabolomics.

**Table 4:** Summary of studies on CC biomarkers discovered through metabolomics

Article type	Population	Study period	Sample size	Source of sample	Platform/assay technique	Stage of research	Association to CC	Significance	References
Research article	China	NA	136 cases 149 normal controls	Plasma	UPLC-MS	Prospective study	Risk/ susceptible marker Diagnostic marker	Bilirubin, LysoPC (17:0), n-oleoyl threonine, 12-hydroxydodecanoic acid and tetracosahexaenoic acid	[84]
Research article	United States	NA	43 cases 43 healthy controls	Urine, cells (cervical swabs)	GC–MS	Case control study	Prognostic marker	5-oxoprolinate, erythronic acid and N-acetylaspartic acid found in urine samples	[87]
Research article	United States	NA	12 LSIL cases 27 HSIL cases 10 ICC cases 18 healthy HPV- controls 11 healthy HPV + controls	Cervicovaginal lavages, cells (vaginal swabs)	LC–MS	Case control study	Prognostic marker Diagnostic marker Therapeutic marker	3-hydroxybutyrate, eicosenoate, and oleate/ vaccenate	[88]
Research article	Korea	2006–2019	97 CIN 60 CC 69 normal controls	Plasma	UPLC-QTOF-MS	Prospective study	Diagnostic marker	AMP, aspartate, glutamate, hypoxanthine, lactate, proline, and pyroglutamate	[86]
Research article	China	2016–2017	90 CSCC cases	Plasma	UPLC-QTOF-MS	Cross-sectional study	Prognostic marker	Phosphatidyl choline (15:0/16:0), phosphatidyl glycerol (12:0/13:0), actosylceramide (d18:1/16:0), D-Maltose, and phthalic acid	[85]
Research article	China	2015–2016	21 CSCC cases 20 CIN II-III cases 11 healthy controls	Uterine cervical tissue	HR-MAS NMR	Case control study	Predictive marker	Elevated levels of LDL, lactate, and alanine and decreased levels of α- and β-glucose, tyrosine, and phenylalanine Decreased levels of isoleucine, methylproline, creatine, acetate, and scyllo-inositol	[89]

### Integrative multi-omics

**Fig. 2:**
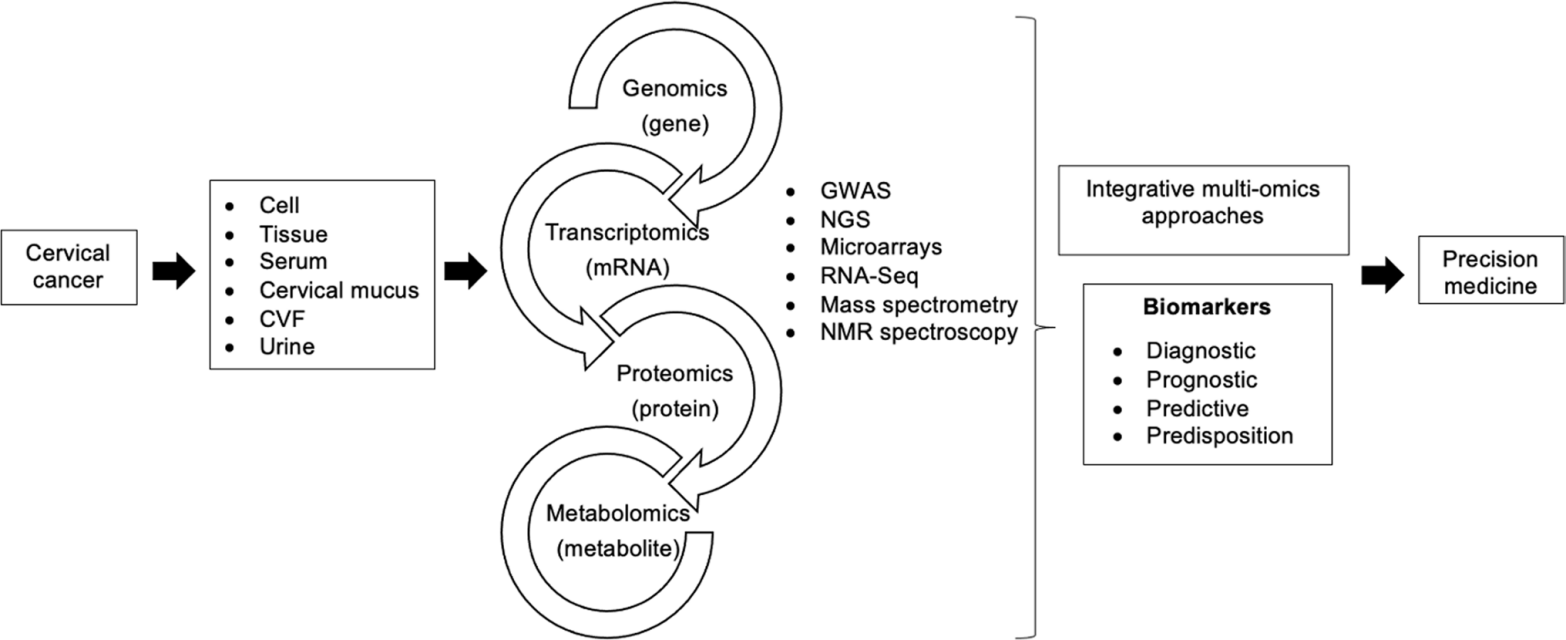
Exploration of cervical cancer biomarkers using omics techniques

**Fig. 3:**
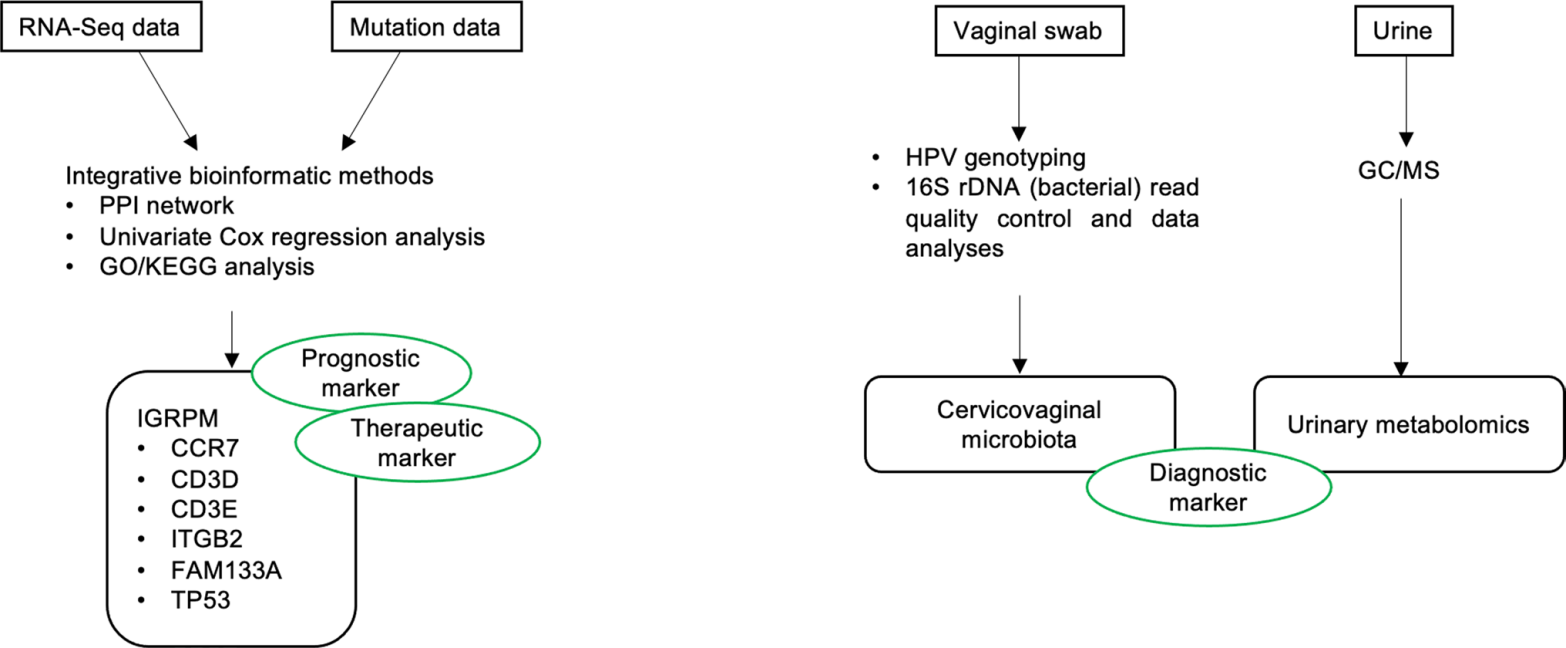
Potential biomarkers identified by the integrative multi-omics analysis

Integrative multi-omics approach (Fig. 2) involving the integration of gene expression profiles with genome-scale biomolecular networks on the CC transcriptomic datasets have revealed the reporter biomolecules at the levels of RNA, protein and metabolite. The potential biomarkers identified by the integrative multi-omics analysis were shown in Fig. 3. Other than the known biomarkers including BRCA1, ESR1, PCNA, FGFR2, CD86, EGFR, P2RX4, ETS1 and E2F4, novel biomolecules including receptors (EPHA4, EPHA5, EPHB2, EDNRA, EDNRB, NCOA3, NR2C1, and NR2C2), miRNAs (miR-192-5p, miR-193b-3p, and miR-215-5p), transcription factors (especially E2F4, ETS1, and CUTL1), other proteins (KAT2B, PARP1, CDK1, GSK3B, WNK1, and CRYAB), and metabolites (particularly arachidonic acids) have been identified as potential biomarkers for the purpose of screening or treatment of CC [12]. Six immune-related genes (chemokine receptor 7 (CCR7), CD3d molecule (CD3D), CD3e molecule (CD3E), and integrin subunit beta 2 (ITGB2), family with sequence similarity 133 member A (FAM133A), and tumor protein p53 (TP53)) identified as prognostic model to forecast the survival and response to immunotherapy to indicate immune status based on multi-omics data analyses [92]. Cervicovaginal microbiome plays a role in hrHPV susceptibility and clearance, and imbalanced cervicovaginal microbiome increases the risk of developing CC [93, 94]. Multi-omics combination of cervical microbiota data with urine metabolomics allows enhanced understanding of community functions in the disease and interactions with host by investigating the association between the host microbiome and circulating metabolites. Other than monitoring compositional changes of bacteria through urine metabolomics, identification of bacteria contributing to the circulating metabolites is also possible through functional characterization of cervicovaginal microbiota and urinary metabolome which may guide the development of diagnostic tools for self-testing [94].

The limited resolving-power for the establishment of casual relationship between molecular signatures and the phenotypic manifestation of cancer hallmarks represents the limitation of single-level omics approaches [95]. On the contrary, investigation of cancer cells or tissues in multiple dimensions by multi-OMICS approaches which investigate cancer may potentially reveal the complicated molecular mechanisms underlying various phenotypes of cancer hallmark, analyze cellular response to treatment as well as contribute to the discovery of clinically relevant biomarkers. Conducting several omics may help to address the challenges arising from the individual use of omics approaches. Integration of omics data is vital for the interpretation of data but challenges arise as it involves computational and/or integration of data or concurrent analysis of multiple variables on multiple datasets [96]. Table 5 shows the summary of studies on integrative multi-omics approaches for CC biomarkers.

**Table 5:** Summary of studies on integrative multi-omics approaches for CC biomarkers

Article type	Population	Study period	Sample size	Source of sample	Platform/assay technique	Stage of research	Association to CC	Significance	References
Research article	NA	NA	306 cases	NA	Integrative multi-omics analysis	NA	Prognostic marker Therapeutic marker	IGRPM comprising six factors, namely CCR7, CD3D, CD3E, ITGB2, FAM133A, and TP53	[92]
Research article	NA	NA	NA	Cells (vaginal and cervical swabs) Urine	Integrative multi-omics analysis	NA	Diagnostic marker	Multi-omic integration of cervical microbiota and urine metabolome	[94]

While biomarkers appear to be potential promising approach to decrease the CC disease burden, they may be too expensive to be applied as viable public health strategy [97]. Despite, in the cost-effectiveness study conducted by Termrungruanglert et al. [97], screening using HPV genotyping test combined with biomarker p16/Ki-67 dual stain cytology as the triage of HPV+ Thai patients aged 30–65 years old is expected to be more cost-effective (average quality-adjusted life years (QALYs) = 24.03, annual cost = $13,262,693) than the Pap cytology (average QALY = 23.98, annual cost = $7,713,251). The improved diagnostic accuracy for CIN2+ of HPV screening with p16/Ki-67 dual stain triage algorithm has enabled higher number of women with precancerous detected and treated in the earlier stages and resulted in lower prevalence and mortality rate [98]. However, the cost of screening, treatment and follow up might be increased due to increased number of patients who return at next screening. The much higher screening costs of the new algorithm had the greatest impact on the total cost [97]. Another study by Juan et al. [99] reported that co-testing (Pap plus HPV mRNA testing including genotyping for HPV 16/18) had greater effectiveness (lifetime QALYs per women screened = 23.01) compared with HPV primary (lifetime QALYs per women screened = 22.99) and lower total costs ($2326 for co-testing v s $2365 for HPV primary) despite the higher screening costs for co-testing.

This study has some limitations such as only online databases were used and there was limited access for some of the published articles. Reviewer and evidence selection bias may occur during screening of studies for the inclusion in this review, and bias may also arise in the primary studies included.

## Conclusions

CC remains a global health issue which require more effective preventive and control strategies [100]. The limitations of current screening and diagnostic strategies for CC prompt the development of novel biomarkers to improve the clinical outcomes of CC patients [20]. In order to benefit the patients, the basic research achievements have to be applied to the clinics. Translational research is used to fill the gap between results of basic research in which biomarkers are discovered and their incorporation into clinical practice [101]. Relatively slow pace of cancer biomarkers being moved into clinical application, which could be attributed to the need of high-performance characteristics for a biomarker to be clinically useful, biology of tumors, inadequacy of the discovery design as well as cumbersome and costly validation process [13]. Regulatory requirements and the lack of reward for translational research also result in the biomarker research to remain stagnant at the discovery phase.

Large scale data provided by high throughput omics technologies has boosted the ability to identify molecular markers of disease processes. Improved patient care can be achieved with co-evolvement of high throughput analyses and biomarker-based precision medicine [20]. Despite, growing gap exists between the big data production and capacity to integrate, process and interpret data. The main challenge faced is to identify which data within the huge data obtained is of clinical relevance, which can be overcome by integrative multi-omics approaches [67]. Collaboration, data sharing, data integration and standards are essential in translating biomarker discovery into clinical use. A global Early Detection Research Network (EDRN) system should be formed to accelerate the pace of biomarker translation. For instance, the US National Cancer Institute (NCI)’s EDRN has been established with four main components, namely: (1) Biomarker Developmental Laboratories (BDLs) for the discovery, development and characterization of new biomarkers or refinement of existing biomarkers, (2) Biomarkers Reference Laboratories (BRLs) for analytical and clinical validation, (3) Clinical Validation Centers (CVCs) which carry out and support biomarker validation trials, and (4) Data Management and Coordinating Center (DMCC) that coordinates network, provides data management and protocol development supporting validation trials as well as conducts related theoretical and applied statistical researches [13]. EDRN aims to foster collaboration between investigators of various expertise and to encourage the rapid movement into clinical validation for successful translational research.

## Data Availability

Not applicable.

## Abbreviations

1D SDS-PAGE: One-dimensional SDS–polyacrylamide gel electrophoresis
ACTN4: Alpha-actinin-4
AIS: Adenocarcinoma in situ
AMP: Adenosine monophosphate
APOA1: Apolipoprotein A-I
BDLs: Biomarker Developmental Laboratories
BRLs: Biomarkers Reference Laboratories
CA19-9: Carbohydrate antigen 19–9
CC: Cervical cancer
CCR7: Chemokine receptor 7
CD44: Cluster of differentiation-44 antigen
CEA: Carcino-embryonic antigen
CIN: Cervical intraepithelial neoplasia
circRNAs: Circular RNAs
CSCC: Cervical squamous cell carcinoma
CVCs: Clinical Validation Centers
CVF: Cervicovaginal fluid
DMCC: Data Management and Coordinating Center
EA: Endocervical adenocarcinoma
EDRN: Early Detection Research Network
ELISA: Enzyme-linked immunosorbent assay
EST: Expressed sequence tags
EVs: Extracellular vesicles
FAM133A: Family with sequence similarity 133 member
GC–MS: Gas chromatography–mass spectrometry
GO/KEGG: Gene Ontology and Kyoto Encyclopedia of Genes and Genomes
GSEA: Gene set enrichment analysis
GSVA: Gene set variation analysis
GWAS: Genome-wide association studies
HLA: Human leukocyte antigen
HPV + H: HPV positive exclusively high-risk
HR-MAS NMR: High-resolution magic angle spinning nuclear magnetic resonance
HSIL: High-grade squamous intraepithelial lesions
ICC: Invasive cervical cancer
IGRPM: Immune gene-related prognostic model
IHC: Immunohistochemistry
ISH: In situ hybridization
ITGB2: Integrin subunit beta 2
LC–MS: Liquid chromatography–mass spectrometry
LC–MS/MS: Liquid chromatography–tandem mass spectrometry
LDL: Low-density lipoprotein
lncRNAs: Long noncoding RNAs
LSIL: Low-grade squamous intraepithelial lesions
MAMA-PCR: Mutation analysis of mismatch amplification PCR
miRNAs: MicroRNAs
MMRN1: Multimerin 1
MPSS: Massively parallel signature sequencing
MRM: Multiple reaction monitoring
MS: Mass spectrometry
NA: Not available
NB: Northern Blot
NCI: National Cancer Institute
ncRNAs: Non-coding RNAs
NGS: Next generation sequencing
NMR: Nuclear magnetic resonance
OR: Odds ratio
ORESTES: Open reading frame ESTs
PCR: Polymerase chain reaction
PPI: Protein–protein interaction
qPCR: Quantitative polymerase chain reaction
RNA-Seq: RNA sequencing
ROC: Receiver operator characteristic curve
RPA: Ribonuclease protection assay
RT-PCR: Reverse transcription polymerase chain reaction
S100A8: S100 calcium-binding protein A8
SAGE: Serial analysis of gene expression
sAng-2: Serum angiopoietin 2
sAng: Serum angiopoietin
SCC-Ag: Squamous cell carcinoma antigen
SCC: Squamous cell carcinoma
SH: Subtractive hybridization
SNPs: Single nucleotide polymorphisms
TCT: Thinprep cytologic test
TP53: Tumor protein p53
QALYs: Quality-adjusted life years
UPLC-MS: Ultra-performance liquid chromatography-mass spectrometry
UPLC-QTOF-MS: Ultra-performance liquid chromatography-quadrupole-time-of-flight mass spectrometry
WGCNA: Weighted gene co-expression network analysis
